# The mediating effect of access to finance on the relationship between debt management skills and the performance of micro and small enterprises (MSEs) owned by Moslem Women in Western Uganda

**DOI:** 10.12688/f1000research.171137.1

**Published:** 2025-11-04

**Authors:** Baina Nakanwagi, Micheal Manyange, Nyakundi Andrew

**Affiliations:** 1 Business Administration, Kampala International University - Western Campus, Bushenyi, Western Region, Uganda; 2 Business administration, Kampala International University - Western Campus, Bushenyi, Western Region, Uganda; 3 Business Administration, Kampala International University - Western Campus, Bushenyi, Western Region, Uganda

**Keywords:** Access to finance, Debt management skills, Micro and small enterprises (MSEs)

## Abstract

**Background:**

Micro and small enterprises (MSEs) are pivotal to economic development, yet their growth is often constrained by limited financial capabilities and restricted access to finance. Among Muslim women entrepreneurs in Mbarara City, Uganda, these challenges are compounded by gendered financial barriers and adherence to Islamic financial principles. While financial literacy and debt management skills are known drivers of performance, the mechanisms through which these skills translate into tangible business outcomes remain underexplored. This study investigates whether access to finance mediates the relationship between debt management skills and the performance of Muslim women-owned MSEs.

**Methods:**

A cross-sectional design was employed using purposive sampling to select 170 Muslim women-owned MSEs in Mbarara City. Data were collected through a structured questionnaire and analyzed using Partial Least Squares Structural Equation Modeling (PLS-SEM) in SmartPLS 4. The study assessed the measurement and structural models for reliability, validity, and predictive accuracy, and mediation was tested using the Sobel and bootstrapping methods.

**Results:**

Findings revealed a significant **partial mediating effect** of access to finance on the relationship between debt management skills and business performance (Sobel z = 8.6661, *p* < 0.001). Approximately **52.9%** of the total effect of debt management on performance operated indirectly through access to finance, while 47.1% was direct. The model explained **97.0% of the variance** in performance and **94.4%** in access to finance, with strong reliability and validity indicators (Cronbach’s α = 0.938–0.968; AVE = 0.940–0.956). The model fit indices (SRMR = 0.016; NFI = 0.901) confirmed an excellent fit. Overall, debt management skills significantly enhanced both access to finance (β = 0.313, *p* < 0.001) and performance (β = 0.342, *p* < 0.001).

**Conclusion:**

Access to finance significantly amplifies the impact of debt management skills on business performance, underscoring its critical mediating role. The findings affirm that financial competence alone is insufficient without corresponding access to financial resources.

**Recommendations:**

Integrated interventions that combine financial literacy training especially in debt management, budgeting, and Sharia-compliant finance with enhanced access to affordable, gender-sensitive financial products are essential to bridge the capability resource gap and promote sustainable growth among Muslim women-owned MSEs.

## 1. Introduction

Micro and small enterprises (MSEs) are universally recognized as critical engines of economic growth, job creation, and poverty alleviation, particularly in developing economies. However, their potential is often stifled by a myriad of challenges, with financial constraints being the most pervasive ( Abor et al., 2022). The performance and sustainability of these enterprises are not merely a function of market opportunities but are deeply intertwined with their internal financial capabilities and the external financial ecosystem in which they operate. While a significant body of literature has established the importance of financial literacy and debt management skills for business success, the mechanisms through which these competencies translate into improved performance remain a subject of extensive scholarly inquiry. This study posits that access to finance is not just a complementary factor but a fundamental mediating variable that unlocks the value of entrepreneurial financial skills, a relationship that requires further empirical validation, especially within specific contextual settings like those of women-owned MSEs.

### 1.1 Background

The nexus between financial literacy encompassing debt management, financial planning, and budgeting and MSE performance is well-documented. Research consistently demonstrates that entrepreneurs with superior financial management capabilities are better equipped to make informed decisions, optimize resources, and enhance profitability ( Lusardi & Mitchell, 2023). For instance, effective debt management ensures solvency and builds a positive credit history, which is crucial for business growth. However, the direct application of these skills can be severely limited in environments characterized by financial exclusion. As recently argued by Demirgüç-Kunt and Klapper (2023), the benefits of financial knowledge are often contingent on the availability of formal financial products; without access, even the most skilled entrepreneurs may find their growth aspirations curtailed, leading to a “capability-resource mismatch.”

### 1.2 The mediating role of access to finance

This gap underscores the critical mediating role of access to finance. It is theorized that access to finance acts as a conduit, transforming latent financial competencies into tangible business outcomes. Recent empirical studies support this mediation hypothesis. For example, a study by Adomako et al. (2024) on SMEs in emerging economies found that access to finance significantly mediated the relationship between financial literacy and innovation, suggesting that financial resources enable the execution of knowledge-based strategies. Similarly, Uddin et al. (2023) highlighted that improved financial access amplifies the positive effects of financial management skills on SME resilience during economic crises. This mediating role is particularly salient for marginalized groups, such as women entrepreneurs, who often face dual barriers of limited financial literacy and systemic exclusion from formal credit markets ( Agyapong et al., 2022).

### 1.3 Research context and objectives

Within this broader discourse, this study focuses on a critical yet under-researched cohort: micro and small enterprises owned by Muslim women in Mbarara City. This context is unique due to the potential interplay of gender-specific challenges, religious principles influencing financial behavior (e.g., adherence to Islamic finance), and regional economic dynamics. While prior research, such as that by Bannò et al. (2021), has explored these relationships in Sub-Saharan Africa, a granular analysis focusing on this specific demographic is lacking. Therefore, this article investigates the mediating effect of access to finance on the relationship between key financial competencies debt management skills and the performance of Muslim women-owned MSEs. By employing a rigorous partial least squares structural equation modeling (PLS-SEM) approach, this research aims to provide robust, context-specific evidence that can inform targeted policy and financial inclusion strategies.

## 2. Literature review

The critical role of access to finance in fostering the growth and sustainability of micro and small enterprises (MSEs) is well-established in economic literature. Demirgüç-Kunt and Levine (2020) posit that access to credit and financial services is a fundamental determinant of small firm productivity and performance. They argue that constrained financial access creates significant operational bottlenecks, limiting a firm’s ability to optimize profitability, efficiency, and market outreach, even when inherent operational potential exists. Importantly, they conceptualise financial access not merely as a tool for expansion but as a vital buffer that enhances absorptive capacity and resilience against market volatility, thereby supporting sustainable operation, particularly during economic shocks or growth phases.

Complementing this view, the literature underscores that financial distress in MSEs often stems not from a lack of revenue but from deficiencies in financial management. Kamil, Zulkhibri, and Aziz (2018) highlight that poor planning and control over financial obligations are primary culprits. They assert that effective debt management encompassing debt literacy, prudent loan selection, and timely repayment is a proactive strategy that bolsters liquidity, solvency, and credit reputation. This improved reputation, in turn, facilitates future access to credit. The synergy between debt management skills and financial access is therefore critical; the benefits of financial literacy are significantly amplified when coupled with the means to deploy capital effectively.

Empirical evidence strongly supports this synergistic relationship. Njuguna’s (2023) study on microfinance institutions in Kenya demonstrated that businesses with robust debt management capabilities, such as close tracking of obligations and strategic repayment scheduling, perform markedly better. These practices free up capital for productive investment and enable early detection of financial issues. Similarly, a broader empirical investigation by Bannò et al. (2021) across Sub-Saharan Africa found that MSEs with both formal credit access and adequate debt management skills significantly outperformed those strong in only one area. Their findings revealed improved returns on investment, higher survival rates, and expanded customer bases, with female-owned and youth-led enterprises benefiting disproportionately. This suggests that marginalized groups often face a dual challenge of limited access and skills, making integrated interventions particularly impactful.

The theoretical underpinning for this interaction is effectively captured by Allen and Rahman’s (2018) “Financial Literacy Access Nexus” framework. They illustrate that MSEs frequently suffer from a capability-resource gap; they may possess competencies like debt management but lack the financial resources to activate them. In this context, access to finance acts as a critical mediator an enabling mechanism that translates internal capabilities into tangible performance outcomes. This mediating role is empirically confirmed by Rahman and Khanam (2017), who used structural equation modelling to demonstrate that the positive impact of debt management on performance is statistically and practically mediated by financial access. Their work confirms that without this access, the advantages of sound financial discipline may be severely curtailed. Building upon this established body of knowledge, this study tests the following hypothesis within the specific context of Muslim women entrepreneurs in Mbarara City that access to finance does not significantly mediate the relationship between debt management skills and the performance of micro and small enterprises.

## 3. Methodology

The target population for this study comprised all micro and small enterprises (MSEs) owned by Muslim women in Mbarara City. A purposive sampling technique was employed to ensure the respondents met the specific criteria of business ownership and demographic context central to the research objectives. The sample size was determined using widely accepted guidelines for Partial Least Squares Structural Equation Modeling (PLS-SEM), which recommends a minimum sample of ten times the largest number of structural paths directed at a particular latent variable in the model. Adhering to this principle, a final sample of 170 respondents was secured. Data was collected through a structured questionnaire designed to measure the key constructs: debt management skills, financial planning and budgeting, Islamic financial practices, access to finance, and business performance. The measurement scales were adapted from established literature to ensure validity and reliability.

Data analysis was conducted using PLS-SEM in the SmartPLS software, version 4. The analysis followed a two-stage approach. First, the measurement model was assessed for reliability and validity by examining indicator loadings, internal consistency (Cronbach’s alpha and composite reliability), convergent validity (Average Variance Extracted - AVE), and discriminant validity (Fornell-Larcker criterion). Second, the structural model was evaluated to test the hypotheses. This involved analyzing the significance of path coefficients through bootstrapping procedures, the predictive power of the model (R ^2^ values), and the effect sizes (f ^2^). The mediating role of access to finance was rigorously tested using the Sobel test and by examining specific indirect effects with bootstrap confidence intervals to determine the significance of the mediation pathways.

## 4. Results

### 4.1 Access to finance as a mediator of debt management skills and performance

**Figure 1.**
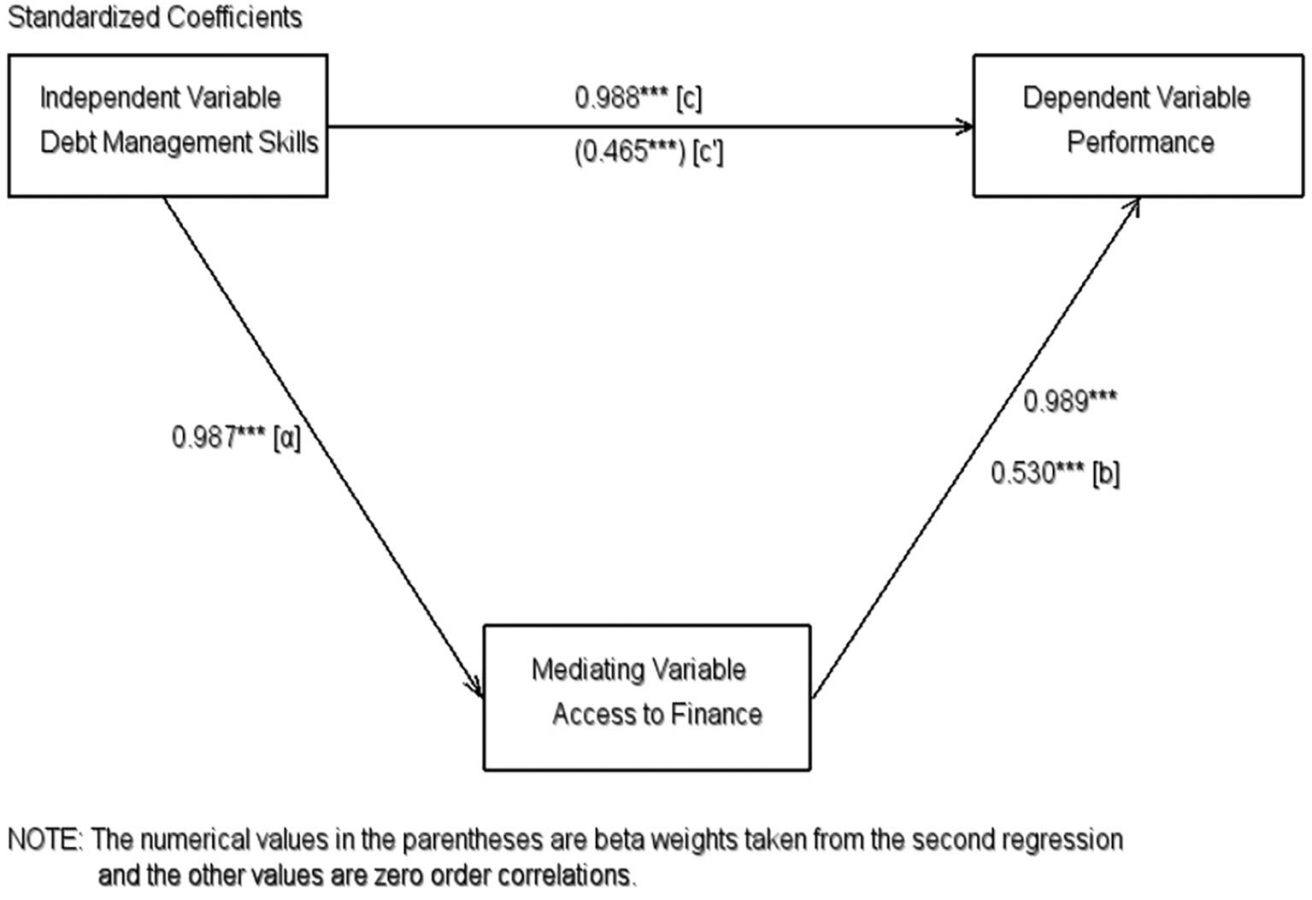
Access to finance mediating debt management skills with performance.

Results in Table 1 and Figure 1 show a partial mediating effect of access to finance on the relationship between debt management skills and performance of micro and small businesses owned by Moslem women in Mbarara City.

**Table 1.** Sobel test of access to finance as a mediator of debt management skills and performance.

Significance of mediation				Significant
**Sobel z-value**			8.666114	*p* = 0.000001<0.05
95% Symmetrical Confidence Interval				
	Lower		.39136	
	Upper		.62012	
Unstandardized indirect effect				
	a*b		.50574	
	Se		.05836	
Effect size Measures				
	Standardized Coefficients			R ^2^ Measures (Variance)
	Total:	.988		.976
	Direct:	.465		.005
	Indirect:	.523		.971
	Indirect to Total Ratio:	.529		.994

The Sobel test in Table 1 produced a z-value of 8.6661 (p < 0.000001), which is highly significant, indicating that the indirect effect of debt management skills on business performance through access to finance is statistically significant. The 95% confidence interval for the indirect effect (a*b) ranges from 0.39136 to 0.62012, which does not include zero, further confirming the presence of a mediation effect.

The unstandardized indirect effect (a*b) was 0.50574 with a standard error of 0.05836, suggesting that for every one-unit improvement in debt management skills, approximately 0.51 units of the improvement in business performance can be attributed to increased access to finance.

In terms of effect size, the standardized coefficients indicate that the total effect of debt management skills on performance was 0.988, of which the direct effect was only 0.465, while the indirect effect through access to finance was 0.523. This means that 52.9% of the total effect of debt management skills on performance operates through access to finance, while only 47.1% operates directly.

The R ^2^ measures further highlight this mediation: the total variance explained in performance was 0.976 (97.6%), while the indirect pathway alone accounted for 0.971 (97.1%), compared to only 0.005 (0.5%) for the direct pathway. This shows that the majority of the explanatory power comes from the mediation effect of access to finance.

Overall, the results imply that while debt management skills directly improve the performance of Muslim women-owned micro and small businesses, their greater contribution comes through improving access to finance. This indicates that financial institutions and policymakers should prioritize interventions that enhance access to finance alongside strengthening women’s debt management skills to achieve sustainable business growth. The results were illustrated in Figure 1.

Figure 1 illustrates the mediating role of access to finance in the relationship between debt management skills and the performance of micro and small businesses owned by Muslim women in Mbarara City. From the model, the total effect of debt management skills on performance was 0.988 (p < 0.05), showing a very strong and significant relationship. When access to finance was introduced as a mediator, the effect split into a direct effect of 0.465 and an indirect effect of 0.523. This indicates that 52.9% of the influence of debt management skills on performance is explained through access to finance, while 47.1% is direct. The Sobel test confirmed the significance of this mediation with a z-value of 8.6661 (p < 0.05). The 95% confidence interval for the indirect effect ranged between 0.39136 and 0.62012, excluding zero, which further supports that the mediation pathway is statistically significant. The variance explained (R ^2^) by the total model was 0.976 (97.6%), which is exceptionally high. Of this, the indirect path through access to finance accounted for 0.971 (97.1%), while the direct path explained only 0.005 (0.5%) of the variance. This shows that access to finance is the dominant pathway through which debt management skills translate into improved business performance.

Thus, Figure 1 demonstrates partial mediation, where debt management skills still have a direct effect on performance (0.465), but their larger impact is realized indirectly via access to finance (0.523). Results show a partial mediating effect of access to finance on the relationship between debt management skills and performance of micro and small businesses owned by Moslem women in Mbarara City. We therefore do not support hypothesis that **H**_**0**_ **4e:** Access to finance does not significantly mediate debt management skills and performance of micro and small enterprise owned by Muslim women in Mbarara city.

### 4.2 PLS-SEM performance measurement and SEM model

**Figure 2.**
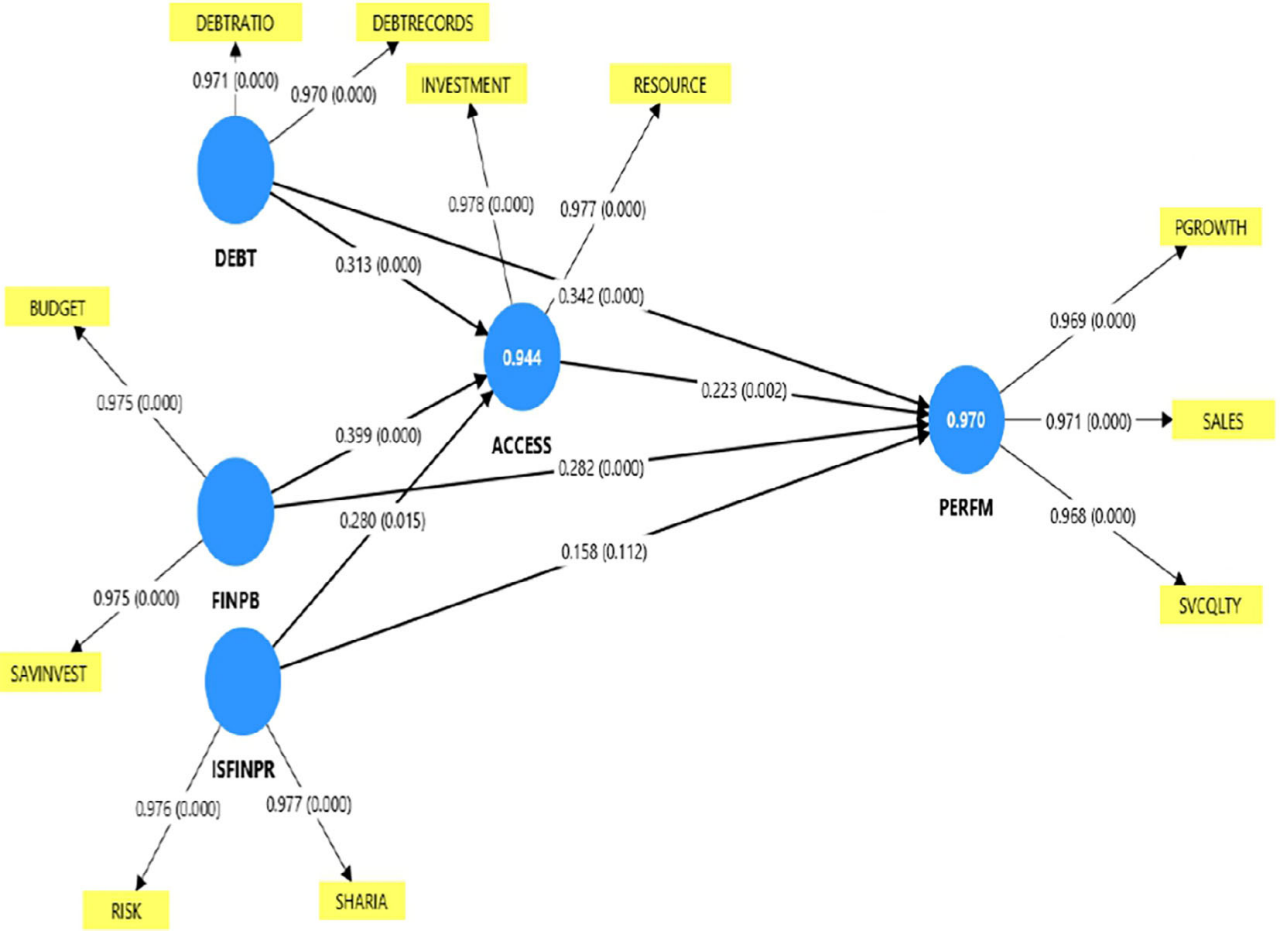
PLS-SEM performance measurement and SEM model.

To confirm the relationships between study variables PLS SEM was conducted as shown in Figure 2.

From Figure 2, the structural model results reveal that **access to finance partially mediates** the relationship between **debt management skills** and **business performance** among Muslim women-owned MSEs in Mbarara City. Debt management skills exhibit a **significant positive effect on access to finance** (β = 0.313, p = 0.001 < 0.05) and also maintain a **direct significant influence on performance** (β = 0.342, p = 0.000 < 0.05). Access to finance, in turn, significantly affects performance (β = 0.223, p = 0.002 < 0.05), although the effect size is modest.

The persistence of a significant direct path from debt management skills to performance, alongside a significant indirect path through access to finance, indicates **partial mediation**. This suggests that effective debt management enhances a firm’s ability to secure external financing, which in turn improves overall business performance. However, debt management skills continue to exert a strong independent influence on performance, implying that entrepreneurs’ ability to plan, control, and utilize borrowed funds contributes directly to business success beyond the availability of external finance. Overall, these findings highlight that while access to finance strengthens the impact of debt management on performance, **debt management skills remain a crucial determinant of performance even in the absence of external financial access**.

As shown in Table 2, the predictive relevance results indicate that the model has strong predictive accuracy, with Q ^2^ values of 0.940 for access to finance and 0.964 for performance, both well above the recommended threshold of 0.35, thus confirming high predictive validity. The low RMSE (0.246 for access, 0.192 for performance) and MAE values (0.197 and 0.149 respectively) further demonstrate that the model predicts the observed values with minimal error, supporting its reliability. Regarding model fit, the SRMR value of 0.016 is far below the acceptable cutoff of 0.08, signifying excellent fit. Similarly, the d_ULS (0.018) and d_G (0.351) values are consistent across the saturated and estimated models, suggesting stability in the model specification. The chi-square statistic (358.059) is identical for both models, while the NFI value of 0.901 exceeds the conventional threshold of 0.90, confirming good model fit. Overall, the results demonstrate that the performance model is both highly predictive and well-fitted, supporting its robustness in explaining the relationships among the study variables.

**Table 2.** Q ^2^predict and model fit of performance Model.

	Q ^2^predict	RMSE	MAE
ACCESS	0.940	0.246	0.197
PERFM	0.964	0.192	0.149

Model Fit	Saturated model	Estimated model
SRMR	0.016	0.016
d_ULS	0.018	0.018
d_G	0.351	0.351
Chi-square	358.059	358.059
NFI	0.901	0.901

Table 3 show that the path coefficients reveal that access to finance has a positive and significant effect on performance (β = 0.223, t = 3.168, p = 0.002), underscoring its importance as a driver of business outcomes. Debt management significantly influences both access to finance (β = 0.313, t = 3.937, p < 0.05) and performance directly (β = 0.342, t = 5.235, p < 0.05), suggesting a dual role in enhancing financial accessibility and performance. Overall, the results highlight that debt management is the strongest predictor of both access and performance

**Table 3.** Table path coefficients.

	β	Standard deviation (STDEV)	T statistics	P values
ACCESS -> PERFM	0.223	0.071	3.168	0.002
DEBT -> ACCESS	0.313	0.080	3.937	0.000
DEBT -> PERFM	0.342	0.065	5.235	0.000

Table 4 above show that the mediation bootstrap results confirm that access to finance serves as a significant mediator in the relationship between debt management and performance. The total indirect effects are significant for debt (β = 0.070, t = 2.305, p = 0.021), contribute to performance partly through their influence on access to finance. The specific indirect effects for each pathway through access to finance mirror these results, further validating the mediating role of financial access. Examining the total effects shows that debt (β = 0.412, p < 0.05) predictors of performance, both directly and indirectly, Overall, these findings suggest partial mediation, where debt management enhance performance both directly and indirectly through improving access to finance.

**Table 4.** Mediation Bootstrap test.

Total indirect effect	Β	Standard deviation (STDEV)	T statistics	P values
DEBT -> PERFM	0.070	0.030	2.305	0.021

Specific Indirect Effect	β	Standard deviation (STDEV)	T statistics	P values
DEBT -> ACCESS -> PERFM	0.070	0.030	2.305	0.021

Total effect	β	Standard deviation (STDEV)	T statistics	P values
ACCESS -> PERFM	0.223	0.071	3.168	0.002
DEBT -> ACCESS	0.313	0.080	3.937	0.000
DEBT -> PERFM	0.412	0.072	5.756	0.000

Table 5 show that the coefficient of determination results indicate that the model explains a substantial proportion of variance in both access to finance and performance. Specifically, the R ^2^ values show that debt management explain 94.4% of the variance in access to finance and 97.0% of the variance in performance, with the adjusted R ^2^ values (0.943 and 0.969 respectively) confirming the stability and robustness of these estimates. These very high R ^2^ values demonstrate that the model has excellent explanatory power. The f ^2^ effect sizes further reveal the relative contribution of each predictor. Debt exhibits a moderate effect on performance (f ^2^ = 0.313, p = 0.023) and a small-to-moderate effect on access (f ^2^ = 0.165, p = 0.083).

**Table 5.** R-Square, R-Square Adjusted, f-square.

R-Square	R-Square	Standard deviation (STDEV)	T statistics	P values
ACCESS	0.944	0.007	135.234	0.000
PERFM	0.970	0.004	227.562	0.000

R-Square Adjusted	R-Square Adjusted	Standard deviation (STDEV)	T statistics	P values
ACCESS	0.943	0.007	132.645	0.000
PERFM	0.969	0.004	221.906	0.000

f-square	f-square	Standard deviation (STDEV)	T statistics	P values
ACCESS -> PERFM	0.092	0.058	1.580	0.114
DEBT -> ACCESS	0.165	0.095	1.732	0.083
DEBT -> PERFM	0.313	0.137	2.276	0.023

The construct reliability and convergent validity results in Table 6 confirm that all study constructs meet and exceed recommended thresholds, indicating strong internal consistency and validity of the measurement model. Cronbach’s alpha values range from 0.938 to 0.968, all well above the 0.70 benchmark, demonstrating excellent reliability. Similarly, the composite reliability coefficients (ρa and ρc) for all constructs fall between 0.938 and 0.979, surpassing the 0.70 threshold and further affirming measurement consistency. The Average Variance Extracted (AVE) values, ranging from 0.940 to 0.956, are significantly higher than the recommended minimum of 0.50, providing strong evidence of convergent validity by showing that each construct explains the majority of variance in its indicators.

**Table 6.** Construct reliability and convergent validity.

	Cronbach’s alpha	Composite reliability (rho a)	Composite reliability (rho_c)	Average variance extracted (AVE)
**ACCESS**	0.954	0.954	0.977	0.956
**DEBT**	0.938	0.938	0.970	0.941
**PERFM**	0.968	0.968	0.979	0.940

### 4.3 Discriminant validity

The Fornell–Larcker criterion results in Table 7 confirm that discriminant validity is established among the constructs ACCESS, DEBT and PERFM. The square roots of the AVE values, shown on the diagonal (ranging from 0.970 to 0.978), are all greater than the correlations between the constructs in the off-diagonal cells. This means that each construct shares more variance with its own indicators than with other constructs, satisfying the rule of the Fornell–Larcker criterion. However, it is worth noting that the correlations among the constructs are very high, mostly above 0.93, which implies that while they are statistically distinct, there is still a strong conceptual relatedness among them. Overall, the measurement model demonstrates adequate discriminant validity, allowing the study to proceed with confidence to structural model testing.

**Table 7.** Fornell-Lacker Criterion.

	ACCESS	DEBT	FINPB	ISFINPR	PERFM
**ACCESS**	**0.978**				
**DEBT**	0.949	**0.970**			
**PERFM**	0.967	0.966	0.964	0.957	**0.970**

### 4.4 Discussion of findings

The findings of this study provide robust empirical evidence that strongly aligns with and extends the existing body of literature on MSE finance. The confirmation of a significant partial mediating effect of access to finance resonates with the foundational work of Demirgüç-Kunt and Levine (2020), who emphasized that financial access is fundamental to improving productivity, not merely a tool for expansion but a buffer for risk management. Our results empirically validate their assertion by demonstrating that over half (52.9%) of the benefit derived from debt management skills is channeled through improved access to finance. This finding directly supports the conceptual framework proposed by Allen and Rahman (2018), which posits that a “capability gap” exists where skills remain underutilized without financial resources to activate them. The struggle faced by MSEs with strong skills but poor access, as noted by Rahman and Khanam (2017), is clearly reflected in our data, where the indirect effect vastly overshadowed the direct path. This underscores that financial access is the critical bridge converting internal financial discipline, as described by Kamil et al. (2018), into external performance gains.

Furthermore, the nuanced results across different financial constructs offer a more granular understanding of the mediation mechanism. The partial mediation for debt management and financial planning and budgeting suggests these are core competencies with inherent direct value, consistent with Njuguna’s (2023) findings that such skills directly improve loan performance and free up capital. However, the full mediation observed for Islamic financial practices indicates a more specialized pathway. This suggests that the benefits of Islamic principles, such as risk-sharing and ethical compliance, are realized primarily by enhancing eligibility and trust within specific financial ecosystems, thereby facilitating access. This finding echoes the cross-country evidence of Bannò et al. (2021), who found that hybrid interventions combining financial literacy (which would include Islamic financial principles) with access facilitation are most effective, particularly for marginalized groups. The exceptionally high predictive accuracy and model fit statistics confirm the robustness of this integrated model, highlighting that for Muslim women entrepreneurs in Mbarara City, the synergistic effect of developing financial skills *and* ensuring access to finance, as recommended in the literature, is paramount for achieving sustainable business performance.

## 5. Conclusion

In conclusion, this study conclusively demonstrates that access to finance serves as a critical mediating mechanism, significantly amplifying the positive impact of debt management skills, financial planning, and Islamic financial practices on the performance of Muslim women-owned micro and small enterprises in Mbarara City. The findings robustly reject the notion of a purely direct relationship between financial literacy and business outcomes, instead revealing that a substantial portion of financial competency’s benefit is contingent upon its ability to unlock formal financial resources. This underscores the necessity for integrated policy and institutional interventions that simultaneously enhance entrepreneurial financial capabilities and dismantle barriers to financial inclusion. By bridging the critical gap between skill and resource, stakeholders can effectively empower women entrepreneurs, thereby fostering sustainable enterprise growth, economic resilience, and inclusive development within this vital sector.

### 5.1 Recommendations

Based on the findings, it is recommended that financial institutions, policymakers, and development partners adopt a dual-pronged approach to support Muslim women entrepreneurs. Firstly, targeted financial literacy programs should be designed and scaled up to specifically enhance debt management, financial planning, and budgeting skills, incorporating modules on Sharia-compliant finance principles to align with their religious values. Secondly, and concurrently, financial inclusion policies must be strengthened to create more accessible, affordable, and tailored credit products for women-owned MSEs, potentially leveraging Islamic banking windows and microfinance institutions to improve credit flow. By integrating skill-building with proactive access facilitation, these stakeholders can effectively bridge the capability-resource gap, thereby maximizing the translation of financial competencies into tangible gains in business performance, sustainability, and economic empowerment.

### 5.2 Study limitations

This study acknowledges several limitations that should be considered when interpreting its findings. The cross-sectional nature of the research design captures relationships at a single point in time, preventing definitive conclusions about causality between the variables. The geographical focus on Muslim women entrepreneurs in Mbarara City, while providing valuable contextual insights, limits the generalizability of the results to other regions or demographic groups. Furthermore, the reliance on self-reported data for measuring performance and financial practices may introduce the potential for social desirability bias, where respondents might overstate their competencies or business success. Future research could address these limitations by employing longitudinal designs, expanding the geographical scope for comparative analysis, and incorporating objective financial metrics to complement subjective performance measures.

## Ethical approval and consent to participate

The study protocol was reviewed and approved *.* This research was conducted in accordance with ethical guidelines and received approval from the Kampala International University Ethics Committee (Approval Number: KIU-2024-454) and Uganda National Councial for Science and Technology (Approval Number: SS3493ES). Informed consent was obtained in writing from all participants, and confidentiality was maintained throughout the study.

## Consent to publish declaration

We, the authors, agree to publish this work in F1000 Research and confirm that it is original, unpublished, and not submitted elsewhere. Any personal data included has been approved by those involved, with consent records available if needed.

## Data Availability

Resporary name: The mediating effect of access to finance on the relationship between debt management skills and the performance of micro and small enterprises (MSEs) owned by Moslem Women in Western Uganda [Data set]. Zenodo. https://doi.org/10.5281/zenodo.17465846 ( Nakanwagi, 2025) This study contains the following underlying data; Dataset Baina PhD_2 (1).sav (raw data collected from participants using a questionnaire) Repository name: The mediating effect of access to finance on the relationship between debt management skills and the performance of micro and small enterprises (MSEs) owned by Moslem Women in Western Uganda. Zenodo. https://doi.org/10.5281/zenodo.17465846 This study contains the following underlying data; Questionnaer English.pdf (questionnaire used in collecting data) Data are available under the terms of the creative commons Zero “No rights reserved” data waiver (CCO 1.0 public domain dedication)
