# Supplementary material for: The mediating effect of access to finance on the relationship between debt management skills and the performance of micro and small enterprises (MSEs) owned by Moslem Women in Western Uganda
Source: F1000Res. 2025 Nov 4;14:1205. [Version 1] doi: 10.12688/f1000research.171137.1 (PMC12949376; doi:10.12688/f1000research.171137.1)
Supplement: Peer review history

# Reviewer report: Reviewer response for version 1

**DOI:** 10.5256/f1000research.188696.r451426

**Published:** 2026-02-28

**Authors:** Jaco Fouche

**Affiliations:** 1 North-West University, Potchefstroom, South Africa

- The literature review presented in Sections 1 and 2 is limited in scope, with several key concepts relying heavily on a single source. This weakens the theoretical grounding of the study and limits engagement with the broader body of existing literature.
- The manuscript does not clearly articulate its original contribution to the literature. The findings appear largely confirmatory and replicate results already established in prior studies, without sufficient justification for why this additional investigation is necessary.
- No motivation or rationale is provided for the selection of the specific sample used in the study, making it difficult to assess the appropriateness and generalisability of the results.
- In Section 4.1, reference is made to on page 6 to: *“We therefore do not support hypothesis H04e.”* However, no hypotheses are explicitly stated or numbered earlier in the manuscript. The notation “H04e” further suggests the existence of multiple hypotheses, none of which are clearly defined or presented.
- There are also inconsistencies in the referencing of tables. For example, the text refers to “Table 4 above” on page 7 ( *“Table 4 above shows the mediation bootstrap results”*), yet Table 4 appears on page 8, following the discussion. This should be corrected for clarity and accuracy.

Is the work clearly and accurately presented and does it cite the current literature?

Partly

If applicable, is the statistical analysis and its interpretation appropriate?

I cannot comment. A qualified statistician is required.

Are all the source data underlying the results available to ensure full reproducibility?

Yes

Is the study design appropriate and is the work technically sound?

Yes

Are the conclusions drawn adequately supported by the results?

Yes

Are sufficient details of methods and analysis provided to allow replication by others?

Yes

Reviewer Expertise:

Personal finance

I confirm that I have read this submission and believe that I have an appropriate level of expertise to confirm that it is of an acceptable scientific standard, however I have significant reservations, as outlined above.

# Reviewer report: Reviewer response for version 1

**DOI:** 10.5256/f1000research.188696.r451431

**Published:** 2026-01-24

**Authors:** Suci Rohayati

**Affiliations:** 1 Universitas Negeri Surabaya, East Java, Indonesia

Although the manuscript demonstrates strong methodological rigor and provides comprehensive statistical analysis, several important issues should be addressed to further strengthen its scientific quality. First, the rationale for focusing specifically on Muslim women–owned MSEs is not sufficiently developed in the introduction; the paper would benefit from stronger empirical or theoretical justification explaining why this group faces distinct financial or institutional constraints compared to other entrepreneurs. Relatedly, the discussion of Islamic financial principles appears only in the later sections, whereas it should be introduced earlier in the literature review to better frame the study’s conceptual foundation. Second, while the analytical approach is sound, the methodology section lacks sufficient detail regarding the measurement instruments, as the indicator variables and scale items are not clearly described or tabulated, which limits the study’s replicability. The absence of an explicit conceptual or structural model diagram in the methods section further reduces methodological transparency. In addition, although the statistical results are robust, the exceptionally high R² values raise concerns about possible common method bias or construct overlap, which should be acknowledged and briefly discussed. Finally, minor editorial issues remain, particularly the inconsistent use of the terms “Moslem” and “Muslim,” which should be standardized throughout the manuscript to ensure academic consistency and clarity.

Is the work clearly and accurately presented and does it cite the current literature?

Yes

If applicable, is the statistical analysis and its interpretation appropriate?

Yes

Are all the source data underlying the results available to ensure full reproducibility?

Yes

Is the study design appropriate and is the work technically sound?

Yes

Are the conclusions drawn adequately supported by the results?

Yes

Are sufficient details of methods and analysis provided to allow replication by others?

Partly

Reviewer Expertise:

entrepreneurship, MSEs, accounting

I confirm that I have read this submission and believe that I have an appropriate level of expertise to confirm that it is of an acceptable scientific standard.

# Reviewer report: Reviewer response for version 1

**DOI:** 10.5256/f1000research.188696.r443925

**Published:** 2026-01-08

**Authors:** Juwita Purnami Restu Suwondo

**Affiliations:** 1 Universitas Merdeka Malang, Malang, East Java, Indonesia

This manuscript investigated excellently how debt management skills influenced the performance of micro and small enterprises (MSEs) owned by Moslem Women in Western Uganda by passing through a third variable, which was access to finance, while focusing on limitations of access for Moslem Women due to cultural and religious principles. Several insights that need to be properly addressed and adjusted accordingly and/or be given clear explanations are the following:

**Introduction:**

- Lacking underlying basis as to why Moslem women-owned MSEs were chosen specifically (lacking previous research, data around the phenomenon that built significance of the chosen topic). Is there any substantial basis for Moslem women-owned MSEs to be lacking financial access or to demonstrate an inadequacy of debt management in some sort?
- Lacking supporting background for the chosen variable of debt management skill instead of investigating access of finance directly as a variable

**Literature Review**:

- Requires more comprehensive theories or previous research covering the need for Moslem women-owned MSEs to be indulged in this study
- Islamic practices were not mentioned in this section (even though it will appear in both Discussion & Conclusion in page 9)

**Methods**:

- I suggest to specify indicator variables for each variable in the model, not only for them to be revealed in Result, but also in Methodology section. The model needs to be displayed as well.

**Discussions**:

- It is not further explored the gravity of the need for Moslem women-owned MSEs to be portrayed in this research specifically, because the findings of the result could be explained without this underlying reason.
- Islamic principles that were mentioned in page 9 were not elucidated beforehand, either in Introduction or Literature Review.

Minor revision: inconsistencies in the writing of "Moslem & Muslim" throughout the manuscript. I suggest to prefer only 1 of them.

Is the work clearly and accurately presented and does it cite the current literature?

Yes

If applicable, is the statistical analysis and its interpretation appropriate?

Yes

Are all the source data underlying the results available to ensure full reproducibility?

Yes

Is the study design appropriate and is the work technically sound?

Yes

Are the conclusions drawn adequately supported by the results?

Yes

Are sufficient details of methods and analysis provided to allow replication by others?

Partly

Reviewer Expertise:

macroeconomics, monetary, local economic development

I confirm that I have read this submission and believe that I have an appropriate level of expertise to confirm that it is of an acceptable scientific standard, however I have significant reservations, as outlined above.
